# *Gtf2ird1*-Dependent Mohawk Expression Regulates Mechanosensing Properties of the Tendon

**DOI:** 10.1128/MCB.00950-15

**Published:** 2016-04-01

**Authors:** Tomohiro Kayama, Masaki Mori, Yoshiaki Ito, Takahide Matsushima, Ryo Nakamichi, Hidetsugu Suzuki, Shizuko Ichinose, Mitsuru Saito, Keishi Marumo, Hiroshi Asahara

**Affiliations:** a Department of Systems BioMedicine, Tokyo Medical and Dental University, Tokyo, Japan; b Department of Orthopaedic Surgery, The Jikei University School of Medicine, Tokyo, Japan; c Department of Systems BioMedicine, National Institute of Child Health and Development, Tokyo, Japan; d Research Center for Medical and Dental Sciences, Tokyo Medical and Dental University, Tokyo, Japan; e Department of Molecular and Experimental Medicine, The Scripps Research Institute, San Diego, California, USA

## Abstract

Mechanoforces experienced by an organ are translated into biological information for cellular sensing and response. In mammals, the tendon connective tissue experiences and resists physical forces, with tendon-specific mesenchymal cells called tenocytes orchestrating extracellular matrix (ECM) turnover. We show that Mohawk (Mkx), a tendon-specific transcription factor, is essential in mechanoresponsive tenogenesis through regulation of its downstream ECM genes such as type I collagens and proteoglycans such as fibromodulin both *in vivo* and *in vitro*. Wild-type (WT) mice demonstrated an increase in collagen fiber diameter and density in response to physical treadmill exercise, whereas in *Mkx*^−/−^ mice, tendons failed to respond to the same mechanical stimulation. Furthermore, functional screening of the *Mkx* promoter region identified several upstream transcription factors that regulate *Mkx*. In particular, general transcription factor II-I repeat domain-containing protein 1 (*Gtf2ird1*) that is expressed in the cytoplasm of unstressed tenocytes translocated into the nucleus upon mechanical stretching to activate the *Mkx* promoter through chromatin regulation. Here, we demonstrate that *Gtf2ird1* is essential for *Mkx* transcription, while also linking mechanical forces to *Mkx*-mediated tendon homeostasis and regeneration.

## INTRODUCTION

All living cells experience mechanical stresses in various forms at both cellular and organ levels (1). The extracellular matrix (ECM) not only provides cells with a physical scaffold but also plays a critical role in development, differentiation, and homeostasis (2). The ECM undergoes constant remodeling, adjusting to physical surroundings and external stresses (3). This environment-dependent remodeling results in intertissue variation depending on the type of forces applied (4, 5). The alteration of the physiological stress that the cells experience, either physical or biochemical, affects the extracellular scaffold construction and its normal biological function (5, 6).

The musculoskeletal system experiences the greatest load and mechanical stress in living organisms (7, 8). However, the type of stress varies according to the role of the tissue, even among the mesenchymal tissues. Tendons and ligaments are fibrous tissues that connect muscle to bone or bone to bone, which require both stiffness and elasticity to withstand and absorb stress from daily activities and exercises. Failure of tendon/ligament results in inflammation, rupture, joint instability, and dislocation. Ruptures often require surgery, and treatment of tendon/ligament disorders often results in prolonged periods of rest, severely affecting activities of daily living (9–11). As tendons and ligaments are similar in components, tendons are often used as autografts in ligament injuries (12). Even after healing, these tissues have poor vasculature and form fibrotic scars with reduced elasticity, increasing the risk of recurrence (13). Inadequate repair resulting in the alteration of normal tension may also lead to degeneration of tendons and ligaments (14). This diminution of tissues after loss of tension may be partly explained by enhanced apoptosis, and although the exact response mechanism to the withdrawal of tension is unclear, it confirms the importance of adequate tension in homeostasis (15). Thus, understanding the mechanisms of tendon/ligament homeostasis is important in order to promote healing and repair. Of the various tendons and ligaments in the body, the Achilles tendon is the largest tendon and also experiences the greatest load in the body (7, 8). Therefore, the Achilles tendon is the ideal candidate for *in vivo* and *in vitro* analysis to assess the transcriptional network of tendon-associated genes and their involvement in mechanosensing.

Tendons consist of fibroblast-like cells, referred to as tenocytes. Type I collagen accounts for the most of the tendon dry mass that forms the collagen fibers and fibrils, with the remaining components consisting of type III collagen, proteoglycans (fibromodulin [Fmod], biglycan, and decorin), glycoproteins (elastin, fibrillin, and tenascins), and other ECM proteins (12, 16). To date, transcription factors Mohawk (Mkx) and Scleraxis (Scx) have been identified as essential transcription factors in tendon development (17–20). While Scx is critical during the embryonic stages, Mkx is thought to be important in both development and maturation into adulthood (18–21). Mkx is a transcription factor responsible for the development of tendons, ligaments, and other ligamentous tissues, and the absence of Mkx results in hypoplastic tendons and ligaments, with decreased collagen fibers (19, 22, 23). Downstream genes of *Mkx* include *type I collagen alpha 1* (*Col1a1*) and proteoglycans that regulate fibril growth such as fibromodulin, decorin, and lumican (19, 20). However, no upstream regulators have yet been identified, and elucidation of Mkx regulators is fundamental to uncovering the complex molecular network of tendon development and homeostasis (24).

Tendons and ligaments are load-bearing organs, and there have been reports of tendon-associated gene expression changes with mechanical loading *in vitro* and *in vivo* (25–27). Adequate *in vitro* cyclic stretching of tenocytes and moderate *in vivo* treadmill exercise have been shown to increase expression of tendon-associated genes such as *Scx*, tenomodulin (*Tnmd*), and *Col1* (27). However, there have been no reports linking *Mkx* with mechanical loading, despite its prolonged expression beyond the developmental stages and its presumed role in tendon/ligament homeostasis. This research investigates the role of *Mkx* in mechanical loading, as well as exploring the upstream regulators of *Mkx*.

## MATERIALS AND METHODS

### Ethical statement.

All animal experiments were performed according to protocols approved by the committee at the Tokyo Medical and Dental University.

### Treadmill.

**FIG 1:**
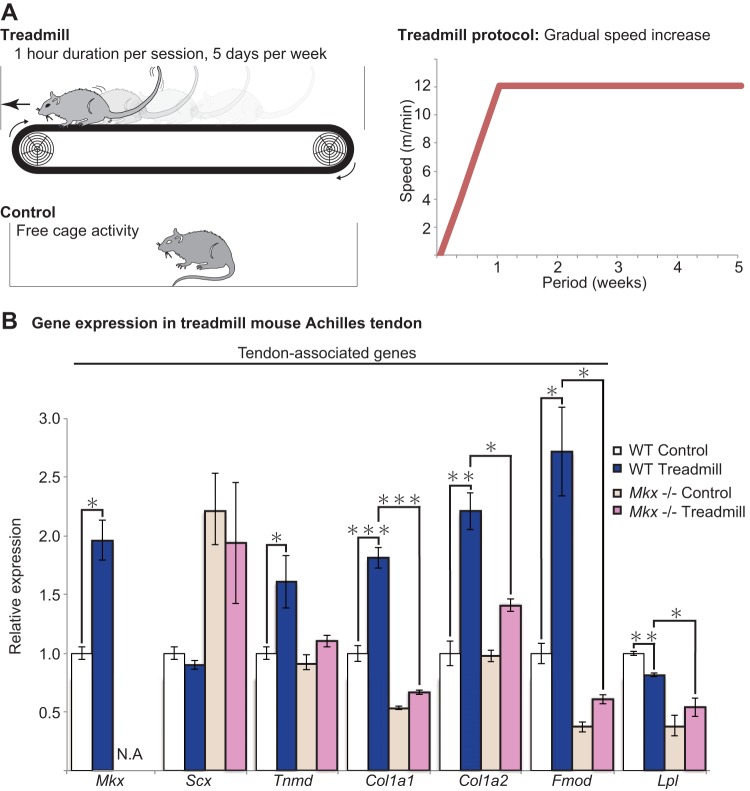
Mechanical loading induces *Mkx* and tendon-associated genes *in vivo*. (A) A schematic illustrating the mouse treadmill experiment and the specific treadmill protocol (right). A control mouse was allowed to move freely within the cage. (B) Tendon-associated gene expression following treadmill exercise. *Mkx*, *Tnmd*, *Col1a1*, *Col1a2*, and *Fmod* were significantly elevated following treadmill exercise. Treadmill exercise in *Mkx*^−/−^ mice results in either no change or only a marginal increase in tendon-associated genes. Lipoprotein lipase (*Lpl*) was selected as a non-tendon-associated gene. Error bars represent standard errors of the means (*n* = 3) (*, *P* < 0.05; **, *P* < 0.01; ***, *P* < 0.001, two-tailed Student's *t* test). N.A., not applicable in *Mkx*^−/−^ mice.

C57BL6/N mice, 10 to 12 weeks old, were placed on a treadmill system (Osaka Microsystem) and were exercised according to their allocated treadmill protocol. Moderate exercise included 12 m/min at 0° inclination for 30 min, 5 days per week, for 4 weeks' duration following a period of acclimatization (Fig. 1A). Three or more mice were used for each RNA analysis. All mice were able to complete their allocated protocol.

### *Mkx*^−/−^ mice.

The *Mkx*^−/−^ mice used were generated and analyzed as described previously (19).

### RNA collection.

RNA collection from rat Achilles tendon was performed after immersion of samples in RNAlater (Ambion). Tissues were cut into 1-mm^3^ pieces and homogenized using a Precellys ceramic beads kit and a Minilys homogenizer (both from Bertin Technologies). RNA collection was performed using a ReliaPrep RNA tissue miniprep system (Promega). RNA collection from cells was performed using a ReliaPrep RNA cell miniprep system (Promega).

### Gene expression analysis by qRT-PCR.

Reverse transcription (RT) was performed using Revertra Ace (Toyobo). Quantitative real-time RT-PCR (qRT-PCR) was performed with Thunderbird Sybr qPCR mix (Toyobo). The expression level of Gapdh was used as an internal control for mRNA expression. Gene expression levels were quantified using the ΔΔ*C_T_* (where *C_T_* is threshold cycle) method. qRT-PCR was performed with three independent samples to confirm reproducibility. Primer sequences for qRT-PCR are listed in Table S1 in the supplemental material.

### Primary rat tenocyte cultivation.

A 3- to 6-week-old Wistar rat was euthanized and briefly immersed in 70% ethanol. An incision was made in the skin, and both Achilles tendons were resected after the surrounding paratenon was removed. The Achilles tendons were cut into 1-mm^3^ pieces and immersed in Triple Express (Gibco) for 30 min before further immersion in Liberase (Roche) for 45 min. The dissolved tissues were filtered before culture at 37°C in 5% CO_2_ in minimum essential medium alpha (MEMα) (Gibco) with 20% fetal bovine serum (FBS) and 1% penicillin-streptomycin. Primary cells were cultured for 5 to 7 days. Medium was changed to MEMα with 10% FBS and 1% penicillin-streptomycin for maintenance. The number of passages of cells used for experiments was kept to fewer than 5 as multiple passages resulted in changes in cell character.

### Cell stretching.

Cells were stretched using an FX-5000 tissue tension system (Flexcell International). Primary tenocytes were trypsinized and seeded onto a type I-collagen-coated chamber and incubated until cell attachment. The cells were stretched at various stretch magnitudes (0.5, 1, 2, 4, 8, and 10%) and frequencies (0.25, 0.5, 1, and 2 Hz) in the incubator at 5% CO_2_ and 37°C. All data shown in this paper represent stretching at 2% and 0.25 Hz for 6 h.

### TEM.

Three Achilles tendon sections of different mice from each protocol were collected at midpoint of the Achilles tendon, midway between the calcaneal attachment and musculotendinous junction, and fixed in glutaraldehyde before dehydration and epoxy resin fixation. Sections were stained with toluidine blue to reveal appropriate tendon structure for transmission electron microscopy (TEM) analysis. Ultrathin slices were obtained and viewed at a magnification of ×50,000. The shortest collagen diameters were counted in three different views (*n* = 100 each) for diameter comparison, and the numbers of collagen fibers per area were determined by counting from three different views to assess fiber density.

### Plasmid construction.

A region 7 kb upstream and a region 3 kb region of the *Mkx* first coding exon were cloned from the C57BL6/N mouse genome and subcloned into a promoterless pGL4.12 luciferase (Luc) vector for screening. Deletion constructs were made by PCR amplification of the respective promoter regions using PrimerSTAR HS DNA polymerase (Clontech); amplified fragments were cut with restriction endonucleases NotI and SalI (Nippon Gene) and then ligated using a DNA ligation kit (TaKaRa). Primers used are listed in Table S2 in the supplemental material. The plasmids were then sequenced to check for mutations.

The *Mkx* upstream region between bp −666 and +319 was further divided into three segments (*Mkx*-del 1, -del 2, and -del 3) and cloned onto a pGL4.12 luciferase vector with a thymidine kinase (*TK*) promoter using EcoRV and BglII restriction enzymes (Nippon Gene) (see Table S3 in the supplemental material). This region was further narrowed down to a 146-bp region (*Mkx*-del 4) by selecting a region of high conservation in mammals. A 68-bp region containing GATTA and GATTA-like motifs was identified within the 146-bp region (*Mkx*-del 4). This 68-bp region was deleted from the *Mkx*-luc 5 vector using inverse PCR (see Table S4 in the supplemental material).

### Functional screening assay.

The 6,049 different expression vectors from the Mammalian Gene Collection (MGC) human cDNA expression vector library were arrayed with pcDNA3.1(+) as a negative control on 384-well plates using 50 ng of plasmid per well. High-throughput transfection assays were performed with 20 μl of Opti-MEM (Gibco) containing 0.1 μl of Lipofectamine 2000 (Life Technologies) and 20 ng of pGL4.12 reporter vector, which were incubated for 20 min. HEK293T cells in 40 μl of medium were added to each well and cultured for 48 h. Luciferase activity was measured using a Steady-Glo luciferase assay system (Promega). A second screening was performed for 35 samples that showed increased luciferase activity in 96-well plates. A third screening was performed in a 24-well plate. Candidate genes were selected based on significant luciferase activity increases when empty luciferase vector without the *Mkx* promoter was used as a control.

### Immunocytochemistry.

Cells were washed with phosphate-buffered saline (PBS) and fixed with 4% paraformaldehyde (PFA) for 15 min at room temperature and rewashed with PBS. Cells were immersed in Blocking One (Nacalai Tesque) for 60 min before the addition of primary antibodies overnight. The samples were then rinsed, and secondary antibodies were added for 1 h before visualization. The primary antibodies used were anti-Gtf2ird1 (AV33735; Sigma) for endogenous Gtf2ird1 (general transcription factor II-I repeat domain-containing protein 1) and anti-FLAG antibody (2368S; Cell Signaling) to detect exogenous FLAG-GTF2IRD1. The secondary antibody used was Alexa Fluor 488 anti-rabbit IgG (A21206; Life Technologies). Hoechst (H3570; Life technologies) was used to stain the nucleus.

### Western blotting.

Protein extracts were collected using radioimmunoprecipitation assay (RIPA) buffer, separated in SDS-PAGE running buffer, and transferred onto polyvinylidene difluoride (PVDF) membrane. Membranes were washed in Tris-buffered saline plus Tween (TBST) and blocked using Blocking One for 1 h. Anti-Gtf2ird1 (1/250) (AV33735; Sigma) was used as the primary antibody at 4°C overnight. Anti-β-actin antibody (1/250) (AC-74; Sigma) was used as an internal control, while anti-H3 antibody (1/2,000) (4499S; Cell Signaling) and anti-α/β-tubulin antibody (1/1,000) (2148S; Cell Signaling) were used as nuclear and cytoplasmic controls, respectively. Anti-rabbit and anti-mouse horseradish peroxidase (HRP)-conjugated antibodies (both at 1/2,000) were used as secondary antibodies (NA9340V and NA931V, respectively; GE Healthcare). Detection reagent (Thermo Scientific) was used to detect the target protein. Stripping solution (Wako) was used for stripping membranes.

### Knockdown experiments using siRNA.

A small interfering RNA (siRNA) was transfected with Lipofectamine RNAiMax (Invitrogen) into primary rat tenocytes. Three Mission siRNAs (Rn_Gtf2ird1_9273; Sigma) for rat *Gtf2ird1* were purchased, and similar results were obtained for all siRNAs (see Fig. S1 in the supplemental material). siRNA 00039274 was used in all experiments. For an siRNA targeting *Mkx* (si*Mkx*), Silencer Select (s145551; Ambion) was used. siRNA (0.03 nmol) was transfected with 6 to 9 μl of RNAiMax and incubated in culture medium for 36 to 48 h before collection and qRT-PCR analysis.

### Luciferase assay.

An *Mkx* promoter-firefly luciferase reporter gene construct (50 ng), an effector gene construct (50 ng), and 5 ng of the pGL4.74 Renilla luciferase construct for normalization (Promega) were cotransfected per well using Fugene HD (Roche) at 50% confluence. Cell extracts were prepared 36 to 48 h after transfection, and luciferase activity was measured using a Dual-Luciferase reporter assay system (Promega).

### Chromatin immunoprecipitation (ChIP).

Primary rat tenocytes, cultured as described above, were transfected with the GTF2IRD1 expression vector from the MGC library with three volumes of polyethylenimine Max (Polysciences, Inc.) and incubated in a 15-cm culture dish for 36 to 48 h before fixation in formaldehyde solution. Sonicated DNA fragments were enriched by immunoprecipitation with rabbit polyclonal anti-Gtf2ird1 antibody (AV33735; Sigma). IgG rabbit antibody (Sc-2027; Santa Cruz) was used as a control. Immunoprecipitated and input DNAs were analyzed by qRT-PCR using primers designed upstream in intervals up to 7 kb (see Table S5 in the supplemental material). Anti-histone H3 trimethylated at K4 (anti-H3K4me3) (ab8580; Abcam), anti-acetyl histone H4 (anti-H4ac) (06-598; Upstate), and anti-RNA polymerase II (Pol II) (8WG16; Covance) were used for histone and Pol II markers.

### Statistical analysis.

A two-tailed independent Student's *t* test was used to calculate the *P* values.

## RESULTS

### Mechanical loading induces *Mkx* and tendon-associated genes *in vivo*.

The effects of mechanical loading were assessed using a mouse treadmill model (Fig. 1A). After a period of acclimatization, moderate treadmill exercise for 4 weeks resulted in an increase in *Mkx* and its downstream genes *Tnmd*, *Col1a1*, *Col1a2*, and *Fmod* but not *Scx* or Lipoprotein lipase (*Lpl*), which was selected as a non-tendon-related gene. (Fig. 1B). The results indicate that under certain conditions, mechanical loading has a positive effect on *Mkx*-mediated tendon gene expression, despite the intricate nature of tendon mechanosensing and strict conditions of tenogenic response.

The novel finding that *Mkx* expression is enhanced by mechanical exercise and the increase in tendon-associated genes independently of *Scx* prompted further investigation to assess whether the same exercise in *Mkx*^−/−^ mice results in similar gene expression changes. The same treadmill protocol for 4 weeks resulted in only a mild increase in *Tnmd*, *Col1a1*, *Col1a2*, and *Fmod* expression levels, which were marginal compared to the increase observed in the wild-type (WT) mouse. The results demonstrate that Mkx is an essential factor for normal tendon response to mechanical stimulation.

### *Mkx*-deficient tendon fails to respond to mechanical stimuli.

**FIG 2:**
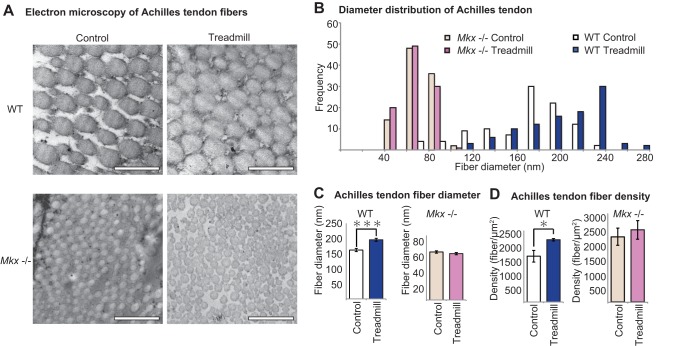
*Mkx*-deficient tendon fibers fail to respond to mechanical loads. (A) Transmission electron microscopy (TEM) of mouse Achilles tendon. WT and *Mkx*^−/−^ collagen fibers from mice with or without exercise are shown. Magnification, ×50,000; scale bar, 500 nm. (B) The collagen fiber diameter distribution graph shows an increase in the distribution of WT mouse fibers but no change was observed in *Mkx*^−/−^ mice. (C) Mean collagen fiber diameter demonstrated an increase in collagen fiber diameter with treadmill exercise for WT mice but not in *Mkx*^−/−^ mice. Data were calculated from three different views (each, *n* = 100). Error bars represent standard errors of the means (***, *P* < 0.001, two-tailed Student's *t* test). (D) Achilles tendon fiber numbers were calculated per area, revealing increased fiber density in the treadmill group for WT mice which was, again, not observed in *Mkx*^−/−^ mice. Data were calculated from three different views (each, *n* = 100). Error bars represent standard errors of the means (*, *P* < 0.05, two-tailed Student's *t* test).

In order to assess whether the altered gene expression of *Col1a1*, *Col1a2*, and *Fmod*, the components of tendon ECM, is reflected at the tissue level, transmission electron microscopy (TEM) was performed in WT and *Mkx*^−/−^ mice with and without treadmill exercise. Transverse Achilles tendon sections revealed an increase in collagen fiber diameter after treadmill exercise in WT mice (Fig. 2A and B) (28). However, in the *Mkx*^−/−^ mouse, not only did the Achilles tendons display reduced collagen diameters, but collagen fiber in the absence of *Mkx* also failed to increase in size in response to physical exercise (Fig. 2C). This suggests that *Mkx* is involved not only in tendon development but also in the tendon response system to physical stimulation that is necessary for the formation of proper tendon fibers.

As *Mkx* is also a transcriptional regulator of proteoglycans such as fibromodulin and decorin, which are involved in forming collagen cross-links, thereby determining the distance between collagen fibers, we calculated the density of collagen fibers (29–31). Collagen fiber densities were not comparable between the WT and *Mkx*^−/−^ mice as collagen fibers of *Mkx*^−/−^ mice had much smaller diameters and were physically able to bundle together more easily. However, when collagen fiber densities were compared between control and treadmill groups, there was a significant increase in fiber density after exercise in the WT group but not in the *Mkx*^−/−^ group (Fig. 2D). This suggests that *Mkx* is again a key component in promoting the bundling of collagen fibers in response to mechanical stimulation, likely through enhanced cross-linking by proteoglycans (32).

### Mechanoforces induce *Mkx in vitro*.

**FIG 3:**
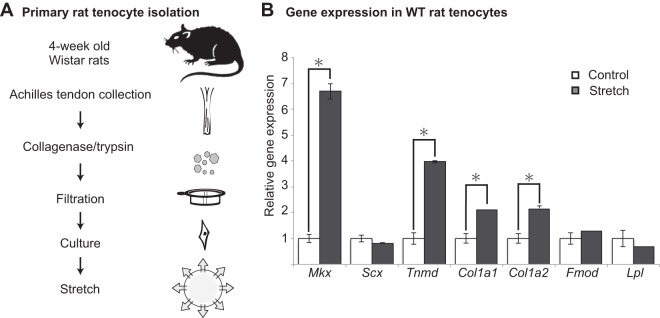
Mechanical stretching induces *Mkx* in primary rat tenocytes *in vitro*. (A) Protocol for obtaining primary rat tenocytes. Tenocytes before passage 5 were used for experiments. Isolated tenocytes were seeded onto collagen-coated chambers and incubated overnight before stretching at 2% at 0.25 Hz for 6 h. (B) *Mkx* and the downstream tendon-associated genes *Tnmd*, *Col1a1*, and *Col1a2*, but not *Scx*, were elevated as a result of tenocyte stretching under specific conditions. Error bars represent standard errors of the means (*, *P* < 0.05, two-tailed Student's *t* test).

Physical forces induced *Mkx* and tendon-related genes *in vivo*. In order to assess the role of *Mkx* at the cellular level, Achilles tendon-derived primary rat tenocytes were subjected to cellular stretching (Fig. 3A). In primary rat tenocytes, *Mkx* induction was most prominent when the tenocytes were stretched using a 2% sinusoidal wave pattern at 0.25 Hz cyclic strain for 6 h. The expression levels of ECM genes downstream of *Mkx*, such as *Tnmd*, *Col1a1*, and *Col1a2*, were also increased, indicating that ECM genes are also responsive in this particular experimental protocol (Fig. 3B). However, *Scx*, another tendon marker that has been reported to have sensitivity to stretching, did not increase. These results show that tenocytes are load-sensitive mechanosensors, inducing *Mkx*-mediated tendon marker expression under specific conditions.

### Functional screening of transcription factors regulating *Mkx* promoter activity.

**FIG 4:**
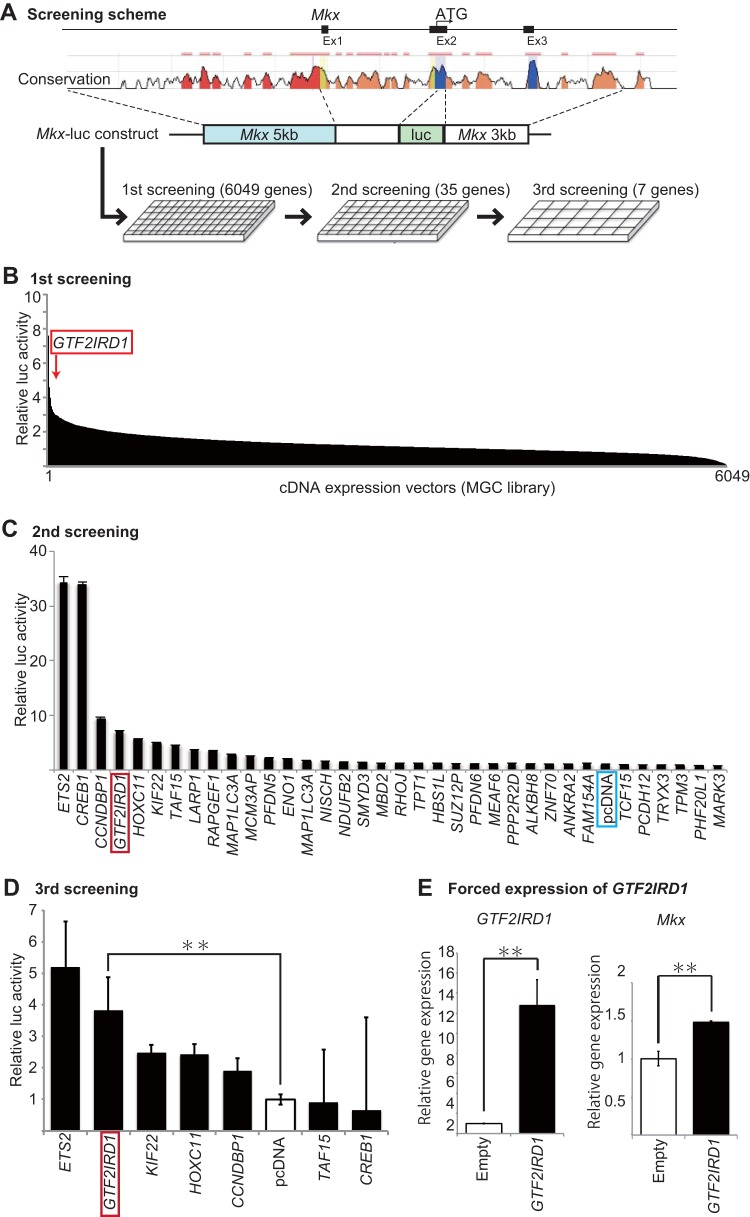
Functional screening for *Mkx*-regulatory genes. (A) A schematic of the screening system. A fragment 5 kb upstream of the transcription start site in addition to the first exon and intron, with a 3-kb region downstream of the first coding exon, was selected and cloned into a luciferase vector. This vector was cotransfected into HEK293T cells with expression vectors for a luciferase assay, which was repeated and narrowed down for a larger-scale analysis. The conservation plot was obtained using the ECR Browser (http://ecrbrowser.dcode.org/) (73). (B) First screening of 6,049 expression vectors performed in 384-well plates (*n* = 1). Thirty-five candidate genes with the greatest increases in luciferase activity were selected for a second screening. (C) Results of the second screening performed in 96-well plates (*n* = 2). Seven genes with consistent luciferase activity increases were selected for a more detailed analysis. Error bars represent standard errors of the means. (D) Results of the third screening performed in 24-well plates (*n* = 2). *ETS2*, *GTF2IRD1*, *KIF22*, *HOXC11*, and *CCNDBP1* were found to elevate luciferase activity in the presence of an *Mkx* promoter. Error bars represent standard errors of the means (**, *P* < 0.01, two-tailed Student's *t* test). (E) qRT-PCR of *GTF2IRD1* transfected tenocytes confirmed *GTF2IRD1* expression. *GTF2IRD1* transfected cells also revealed an increase in *Mkx* expression in rat tenocytes. Error bars represent standard errors of the means (**, *P* < 0.01, two-tailed Student's *t* test).

Currently, there are no known molecular factors linking physical forces with *Mkx* induction, nor have any upstream regulators of *Mkx* been identified. Therefore, a functional screening of upstream regulatory factors of *Mkx* transcription was performed with *Mkx* promoter-driven luciferase constructs with an MGC library of 6,049 human genes (Fig. 4A and B). Among them, 619 genes increased luciferase activity by more than 2-fold, whereas 267 genes decreased activity by more than 50%. Among the genes with increased luciferase activity from the first screening, 35 genes which activated the *Mkx* promoter by greater than 3-fold were analyzed for a second screening (Fig. 4C). Then, among the 32 genes which increased luciferase activity, the seven genes with the greatest luciferase activity were selected for a third screening. ETS2 (v-ets avian erythroblastosis virus E26 oncogene homolog 2) and GTF2IRD1 (general transcription factor II-I repeat domain-containing protein 1) showed the greatest relative luciferase activity increases (Fig. 4D) and were therefore selected as the prime candidate genes for further assessment with regard to *Mkx* promoter activity.

To determine whether GTF2IRD1 expression affects *Mkx* expression, qRT-PCR was performed. *GTF2IRD1* transfection confirmed the increase of *GTF2IRD1* and also demonstrated an increase in *Mkx* expression (Fig. 4E).

### Mechanical stretching induces nuclear translocation of Gtf2ird1.

**FIG 5:**
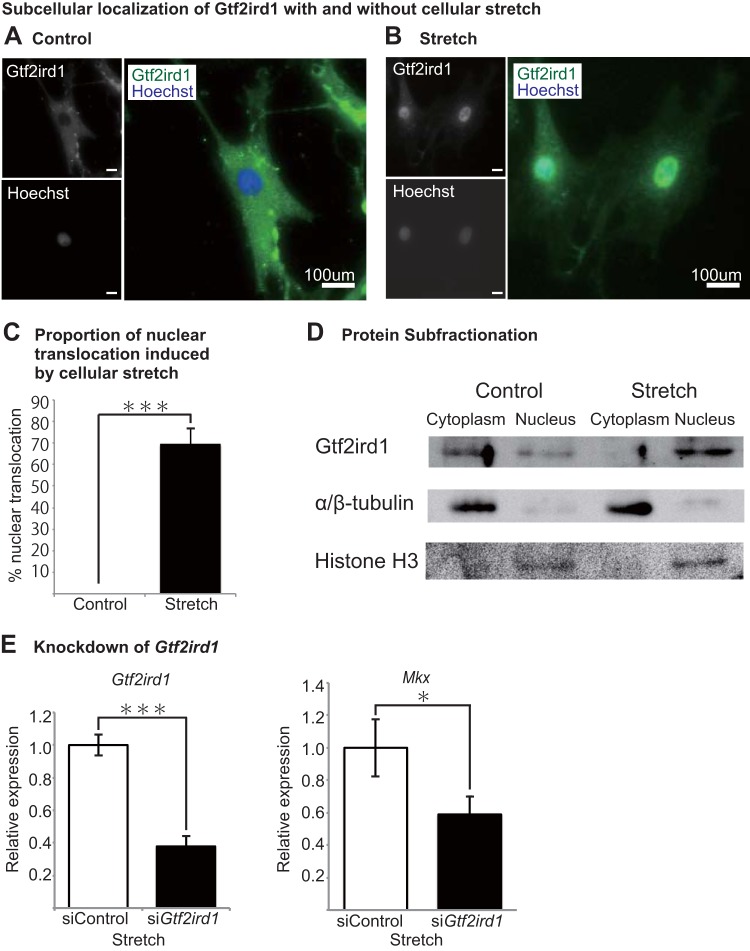
Cellular stretching induces nuclear translocation of Gtf2ird1. (A) Immunocytochemistry of a control rat tenocyte without stretching. Gtf2ird1 was seen predominantly within the cytoplasm in comparison with localization in the nucleus (green, Gtf2ird1; blue, Hoechst). (B) Immunocytochemistry of a rat tenocyte after cellular stretching. Gtf2ird1 expression was most concentrated in the nucleus and also moderately concentrated in the cytoplasm surrounding the nucleus (green, Gtf2ird1; blue, Hoechst). (C) Proportion of cells with nuclear translocation induced by stretching. Error bars represent standard errors of the means (***, *P* < 0.001, two-tailed Student's *t* test). (D) Western blot of fractionated protein extracted from stretched tenocytes confirms that Gtf2ird1 translocates into the nucleus. α/β-Tubulin and histone H3 were used as cytoplasmic and nuclear controls, respectively. (E) *Gtf2ird1* knockdown using siRNA (si*Gtf2ird1*) inhibits *Mkx* expression despite stretching, indicating the importance of *Gtf2ird1* in mechanosensitive *Mkx* regulation. Error bars represent standard errors of the means (*, *P* < 0.05, ***P < 0.001, two-tailed Student's *t* test).

In order to assess whether the candidate genes play a critical role in the mechanical response of tendons, subcellular localization was studied with and without stretching in primary rat tenocytes. Mechanical loading in cells has been shown to induce cell shape changes (27, 33). Here, cyclic stretching resulted in elongated tenocytes that extended perpendicularly to the direction of stretch, as reported previously in mouse embryonic fibroblasts and muscle cells, rat vascular smooth muscle cells, xenopus kidney cells, and bovine aortic endothelial cells (34–37). Immunocytochemistry of Gtf2ird1, in particular, showed significant expression pattern changes as a result of cellular stretching. In cells without stretching, Gtf2ird1 showed cytoplasmic distribution (Fig. 5A). However, tenocytes exposed to mechanical stress revealed nuclear translocation of Gtf2ird1 (Fig. 5B). Almost none of the control tenocytes showed greater Gtf2ird1 expression in the nucleus, whereas more than 80% of stretched tenocytes demonstrated greater expression in the nucleus than in the cytoplasm (Fig. 5C). The proportion of nuclear translocation was also confirmed by Western blotting, where nuclear protein levels increased and cytoplasmic protein levels decreased (Fig. 5D). The result demonstrates that mechanical loading encourages nuclear translocation of Gtf2ird1, indicating that Gtf2ird1 is a mechanosensor in tenocytes.

### *Gtf2ird1* knockdown inhibits *Mkx* increase despite cellular stretch.

To further corroborate the role of *Gtf2ird1* in *Mkx* regulation, we tested the effects of *Gtf2ird1* depletion on *Mkx* expression with mechanical stretching. *Gtf2ird1* knockdown using siRNA did not show increased *Mkx* activity (Fig. 5E) despite the cellular stretching conditions that induce *Mkx* expression in control cells. These results demonstrate the importance of nuclear translocation of Gtf2ird1 in regulating *Mkx* expression in tenocytes.

### Deletion analysis reveals that a GATTA motif-containing sequence is essential for *GTF2IRD1* to regulate *Mkx* activity.

**FIG 6:**
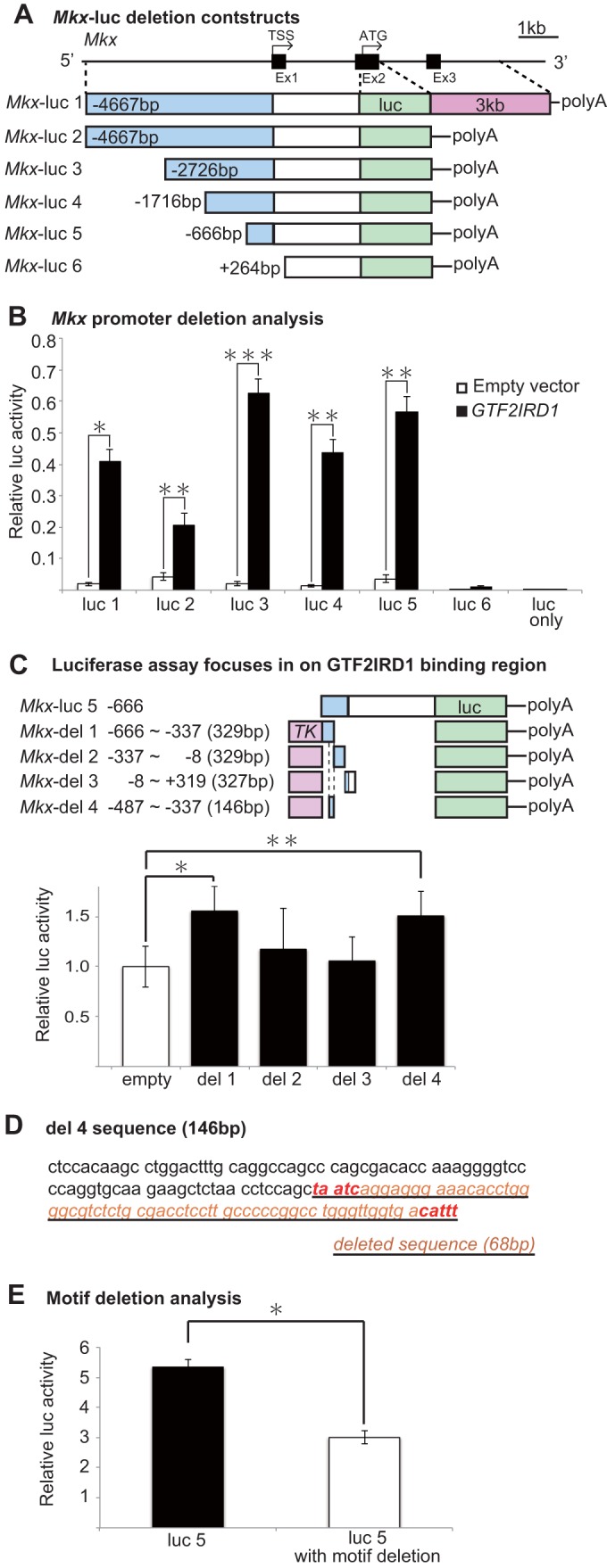
*GTF2IRD1* enhances *Mkx* promoter activity. (A) Schematic for *Mkx*-luciferase deletion constructs. A region 5′ upstream of *Mkx* ATG and a 3-kb downstream region were deleted as shown. TSS, transcription start site. (B) *Mkx* deletion constructs were cotransfected into HEK293T cells with *GTF2IRD1* for luciferase assays. Luciferase activity was severely reduced when the *Mkx* upstream region was shortened to within 666 bp upstream of the transcription start site. Error bars represent standard errors of the means (*, *P* < 0.05; **, *P* < 0.01; ***, *P* < 0.001, two-tailed Student's *t* test). (C) Luciferase assay with shorter constructs with the *TK* promoter narrows down the GTF2IRD1 binding region to 329 bp (*Mkx*-del 1). On closer inspection of the *Mkx*-del 1 sequence, a 146-bp region was highly conserved in mammals. Luciferase assay of this 146-bp region (*Mkx*-del 4) did not show a decrease in luciferase activity compared to that in the *Mkx*-del 1 construct, thus indicating that this 146-bp region is sufficient for the binding of GTF2IRD1. *, *P* < 0.05; **, *P* < 0.01, two-tailed Student's *t* test. (D) A section within the 146-bp *Mkx*-del 4 deletion construct described in panel C reveals a previously described GATTA motif and CATTT, a GATTA-like-containing sequence. A 68-bp region containing these motifs was deleted from the *Mkx*-luc 5 reporter vector for subsequent analysis. (E) Luciferase activity was reduced in the reporter vector with the 68-bp deletion to confirm the importance of the motif-containing deleted region. Error bars represent standard errors of the means (*, *P* < 0.05, two-tailed Student's *t* test).

Having identified GTF2IRD1 as the candidate transcription factor that enhances *Mkx* promoter activity in an *Mkx*-Luc vector containing a 7-kb region upstream and 3-kb region downstream of the first coding exon (exon 2), we generated deletion constructs to narrow down the *Mkx* promoter region that *Gtf2ird1* regulates (Fig. 6A). Luciferase assay of the six deletion constructs revealed low luciferase activity in empty-vector constructs and in the construct containing the 5′ untranslated region (UTR) (Fig. 6B, luc 6). Luciferase activity increased significantly when the deletion constructs were increased to contain a 666-bp region upstream of the transcription start site, suggesting that the critical *Mkx*-regulating region of *Gtf2ird1* exists between bp +264 and −666 upstream of exon 1 (Fig. 6B, luc 5).

In order to further assess the Gtf2ird1 binding region, smaller deletion constructs of ∼300 bp were constructed with a thymidine kinase (*TK*) minimal promoter. A luciferase assay revealed that the region between bp −666 and −337 upstream of exon 1 showed higher luciferase activity than the other regions. Furthermore, a 146-bp sequence within this region was found to be conserved among mammalian species, and this 146-bp sequence alone was sufficient for maintaining luciferase activity (Fig. 6C, *Mkx*-del 4) (38). On examination of this 146-bp region, a previously reported GTF2IRD1 binding sequence, GATTA, and another GATTA-like sequence were found (Fig. 6D). A luciferase assay revealed that deletion of a 68-bp region containing these two sequences is sufficient to cause a decrease in luciferase activity, suggesting that GTF2IRD1 binds to a GATTA motif found in the *Mkx* promoter region (Fig. 6E).

### ChIP-qPCR confirms that Gtf2ird1 binds to the *Mkx* promoter region and is involved in histone modification to regulate *Mkx* transcription.

**FIG 7:**
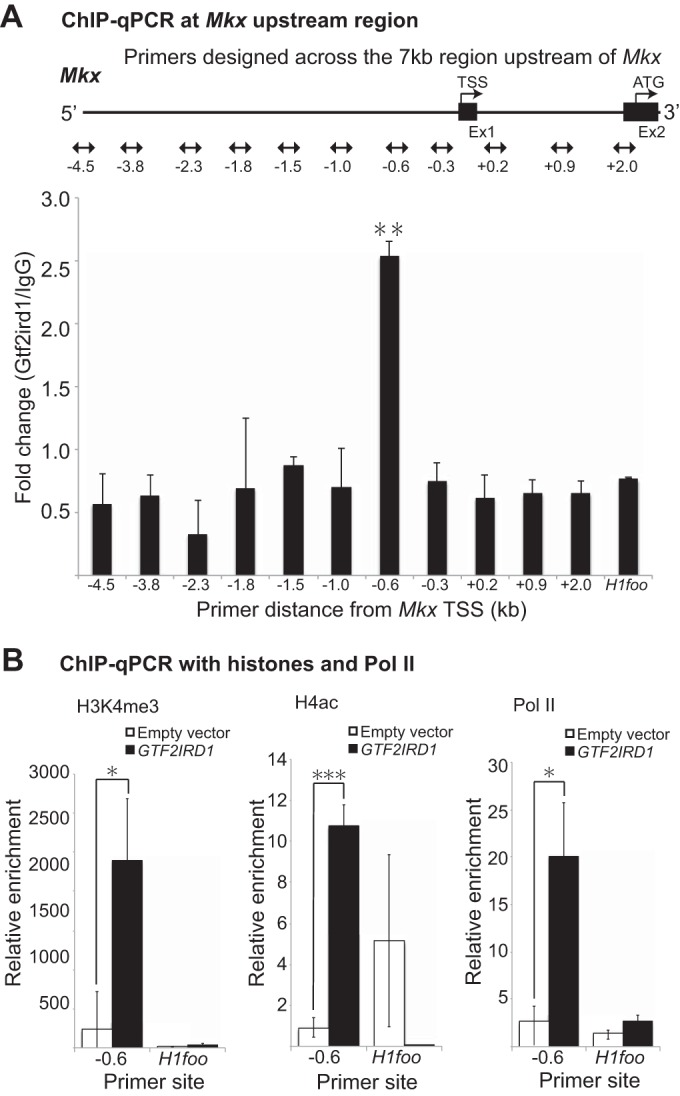
*Gtf2ird1* modifies chromatin at the *Mkx* promoter. (A) ChIP-qPCR analysis of the GTF2IRD1 binding site with anti-Gtf2ird1 antibody using primers designed upstream of the *Mkx* TSS revealed enrichment of DNA at bp −600 upstream (not drawn to scale). Error bars represent standard errors of the means (**, *P* < 0.01, two-tailed Student's *t* test performed for results between primers designed at bp −600 and primers designed at the *H1foo* intron selected as a control). (B) ChIP-qPCR with histones and Pol II at the putative GTF2IRD1 binding site. Anti-H3K4me3, anti-H4ac, and anti-Pol II antibodies were used to assess active histone modification, which confirmed enrichment at the GTF2IRD1 binding site identified from the experiment shown in panel A. Error bars represent standard errors of the means (*, *P* < 0.05; ***, *P* < 0.001, by a two-tailed Student's *t* test).

Having identified the promoter region that Gtf2ird1 acts upon, chromatin immunoprecipitation (ChIP) analysis was performed to assess whether Gtf2ird1 binds to the *Mkx* promoter. The results demonstrate a 2.5- to 5-fold increase of immunoprecipitate in the primer designed around a region 600 bp upstream compared to the amount with the surrounding primers and a negative control (Fig. 7A). This region, which showed the greatest enrichment, was consistent with the finding from the luciferase assay. Furthermore, ChIP-quantitative PCR (qPCR) with H3K4me3, H4ac, and Pol II antibodies revealed enrichment compared with levels with the negative control, demonstrating chromatin modification at this location (Fig. 7B).

## DISCUSSION

Living organisms and, in particular, the motile organs that resist tension such as tendons and ligaments, input physical forces as extrinsic cues obtained from the environment. The molecular mechanisms of how a tenocyte or a ligamentocyte receives the mechanosignals, however, are not fully understood. We investigated the role of Mkx, a tendon-specific transcription factor, in the mechanosensing mechanism of the tendon cell and of the tendon tissue. *Mkx* expression was sensitive to mechanical cues both *in vitro* and *in vivo*, where *Mkx* was induced by cellular stretching in primary rat tenocytes and by physical exercise in the Achilles tendon tissues of mice. The functional screening with transcription factors identified *GTF2IRD1* and *ETS2* as candidate upstream regulators of *Mkx* transcription. Among them, Gtf2ird1 was induced to translocate into the nucleus by the cellular stretching in primary rat tenocytes and was indispensable for stretch-induced *Mkx* upregulation. These findings illuminate a transcriptional network instigated by mechanical forces that converges in *Mkx*, an essential gene for the development of collagen fibers and formation of proper tendons.

### *Mkx* mechanobiology.

The Achilles tendon is the largest load-bearing tendon in the human body, and its molecular network has been investigated in the past using animal models (9, 39). Previous reports have revealed that various tendon-associated genes, such as *Scx* and *Egr1/Egr2* (*Egr1/2*) are responsive to mechanical stimulation (40). *Scx* expression was increased after stretching in bioartifical tendons composed of mouse mesenchymal stem cells, and treadmill running promoted *Scx* expression in the epitenon of mouse Achilles tendon while also enhancing expression in the Achilles tendon itself (26, 27). Egr1/2 have also been implicated in short treadmill exercise of rats, but they are not specific to tendons and are general transcription factors that are involved in chondrogenesis and osteogenesis (41–43).

Mkx is a transcription factor specifically expressed in the developing tendon and is essential for the proper tendon development as shown by the tendon-defects observed in *Mkx*^−/−^ mice (19, 44). How *Mkx* expression is regulated, however, is not well understood. This study shows that mechanical stimulation is a cue that induces *Mkx* expression in tenocytes and in tendon tissues. Tenocytes and tendons receive mechanical forces immediately after birth, during growth, and throughout life. It has not been understood, however, how precisely the mechanical signals that the tendon receives may affect tendon development, homeostasis, or repair.

This study reveals that Mkx, a tendon-regulating transcription factor, is responsive to mechanical stimulation both *in vitro* and *in vivo*. Adequate mechanical stimulation promotes collagen fiber thickening, as observed by TEM, along with increased fiber density. However, this is not observed in *Mkx*-deficient mice, suggesting that *Mkx* is important in collagen fiber development in response to mechanical stimuli. Small leucine-rich proteins (SLRPs) such as fibromodulin and decorin, which are reduced in the absence of *Mkx* (19, 20), may account for the apparent resistance to collagen fiber density increase in *Mkx*^−/−^ mice. SLRPs have been implicated in cross-linking between collagen fibers, and their decrease may prevent normal cross-link formation despite mechanical stimulation (31, 45).

However, tendons and tenocytes are extremely sensitive to mechanical stimuli, and tenogenic genes are elevated when only an appropriate amount of stimulation is applied. Mild stretching of tenocytes *in vitro* increased *Mkx* and tendon-associated genes, but increases in the stretching percentage and frequency resulted in a decrease of tendon markers. This was also observed *in vivo*. For example, short-duration treadmill exercise decreases tendon marker expression, and intense treadmill exercise also inhibits the increase of tendon markers (27). The optimum stretch condition differs from previous studies; however, this is likely to be dependent on interspecies variation and the different devices used (27, 42, 46). Indeed, replication of the tendon marker increase was confirmed with a different stretch device (STB-140; STREX) under slightly different conditions (data not shown). However, excessive cellular stretching decreases *Mkx* and tendon-associated markers, as reported previously (27).

Extreme stretching and intense exercise have been reported to increase osteogenic and chondrogenic marker expression levels in tendons (26, 27). It has also been reported that the nature of mechanical loading affects the stiffness of tendons, with stochastic strains more likely to cause microdamage and decreased stiffness than cyclic strains (47). The nature, amplitude, frequency, duration, and period of mechanical loading are all part of a delicate balance for tendon homeostasis, and our findings imply the sensitive nature of tendons and tenocytes to mechanical signals.

### Transcription factors that regulate *Mkx*.

*Mkx* is specifically expressed in developing tendons, but its transcriptional regulation remains to be elucidated (24). Uncovering the genetic network upstream of *Mkx* would reveal the process of how the tendon lineage is determined during developmental specification. In addition to this, revealing the regulatory network of the mechanosensitive *Mkx* gene would provide insight into the mechanotransduction pathway in tendons (24). Functional screening performed in this study which covered 6,049 genes from the MGC library has identified *GTF2IRD1* and *ETS2* as candidate transcriptional regulators of *Mkx*. In particular, detailed analysis by luciferase assay and ChIP-qPCR assay revealed that GTF2IRD1 regulates *Mkx* activity by binding to the *Mkx* promoter ∼600 bp upstream of the first coding exon. Sequence analysis determined that a previously reported GTF2IRD1 binding motif and a sequence similar to the reported motif exist in the short sequence identified by the luciferase assay. Deletion of these sequences resulted in reduced luciferase activity (Fig. 6D and E) (38). These findings indicate that GTF2IRD1 binds to the GATTA motif in the *Mkx* promoter region to regulate transcription.

*GTF2IRD1* is one of 28 genes that are located in 1.5-Mb region on chromosome 7q11.23 that is commonly deleted in Williams syndrome (48). Whole-mount *in situ* hybridization (WISH) and transgenic mouse staining have shown that *Gtf2ird1* expression appears from embryonic day 8.5 (E8.5) and stains more distinctly in somites and neural crest derivatives at E9.5 before localizing into limbs, tail, and frontonasal primordia (49, 50). This staining pattern of the limbs is in keeping with *Mkx* expression, which appears in the later embryonic stages (19). *Gtf2ird1* null mice showed hemorrhage in the head and body with abnormally developed blood vessels and presents with embryonic lethality between E8.5 and E12.5 (50). Williams-Beuren syndrome (WBS) is caused by the hemizygous deletion of this *GTF2IRD1*-containing region and presents with various musculoskeletal features and neurodevelopmental features (51). Spinal deformities such as kyphosis and scoliosis, as well as joint contracture or laxity, have been reported in humans and in mice (50, 52–54). One of the characteristics of WBS is the malformation of the ascending aorta, which is caused by the haploinsufficiency of the adjacent elastin gene, and its diverse severities and phenotypes may be associated with the concordant deletion length (53, 55). Aortic development is also a mechanoforce-directed morphogenesis (56), and it may be that *GTF2IRD1* has a role in mechanosignal transduction in various tissues, including the vascular system, contributing to the various degrees of severity of this complex syndrome.

In tendons, we show that Gtf2ird1 translocates into the nucleus upon cellular stretching, thereby revealing how the stretch signals induce *Mkx* expression. Recent reports have identified possible proteins interacting with GTF2IRD1, which included transcriptional regulating proteins such as heterochromatin protein 1 (HP1) and specificity protein 1 (SP1) (57). GTF2IRD1 showed some colocalization with zinc finger MYM type 2 (ZMYM2) and ZMYM3, which may implicate their involvement in histone deacetylase (HDAC)-containing silencing complexes. Interestingly, their localization patterns were also similar to the localization of general transcription factor II-I (GTF2I or TFII-I), a member of the GTF2I family that is also deleted in WBS. However, these data are based on HeLa cells, which normally show nuclear localization of GTF2IRD1, and therefore may have different functions in tenocytes, where they reside predominantly in the cytoplasm without external stimulation. GTF2IRD1 may also interact with HDAC3 and protein inactivator of activated STAT xβ (PIASxβ, a member of the E3 ligase family involved in the small ubiquitin-like modifier [SUMO] pathway), also suggesting a role in transcription regulation through alteration of chromatin structures (58). Indeed, histone modification was confirmed at the GTF2IRD1 binding site to show increased histone marks in H3K4me3, H4ac, and Pol II when GTF2IRD1 was overexpressed (Fig. 7B). These findings show that GTF2IRD1 is likely to regulate *Mkx* transcription through chromatin regulation; however, how the stretching-induced nuclear translocation of GTF2IRD1 is molecularly harnessed in relation to other proteins in tendons remains to be elucidated.

### Possible mechanisms of nuclear translocation.

The function of GTF2IRD1 or its regulation of nuclear translocation is still largely unknown and remains an important topic for further research. Possible mechanisms include an ECM-orientated response and phosphorylation. Cytoskeletal changes affect F-actin polymerization and actin binding proteins to induce nuclear shuttling (59). Yes-associated protein (YAP)/transcriptional coactivator with PDZ-binding motif (TAZ) have been of recent interest for their sensitivity to ECM stiffness and actin fiber polymerization and capacity to induce nuclear localization to alter cellular function (60, 61). As YAP/TAZ have a role in mesenchymal cell differentiation, they may also play a part in tendon cell development (62).

Phosphorylation of TFII-I results in the translocation from the cytoplasm to the nucleus and is involved in cell cycle signaling, transforming growth factor β (TGF-β) signaling, endoplasmic reticulum stress response, and immune signaling (63–65). TFII-I has also been shown to interact with TATA-binding protein (TBP)-associated factor 15 (TAF15), one of the other candidates from a functional screening that is known to be associated with gene regulation during cellular stress (Fig. 4D) (66). In order to assess whether the phosphorylation seen in TFII-I is observed in GTF2IRD1, reported phosphorylation sites in TFII-I were searched for in GTF2IRD1 (67–69). TFII-I contained two consensus Src-dependent tyrosine phosphorylation sites with two additional Src homology-binding motifs (67). However, none of the four sequences was found in GTF2IRD1, and this was not pursued further. Despite the lack of common phosphorylation motifs, there still remains the possibility of GTF2IRD1 phosphorylation; therefore, this remains an area to be further explored.

### Mechanoforces are converged in the *Mkx*-oriented transcriptional network.

Mechanoforces converge in *Mkx* induction, but the physiological role of *Mkx* under tenuous conditions is not completely understood. The findings indicate that mechanical cues are essential in collagen fibrillogenesis and also collagen cross-link formation. Furthermore, our recent findings revealed that *Mkx* transfected mesenchymal cells express numerous tendon-related extracellular matrix components, such as collagens and proteoglycans (70). This implies that *Mkx* induces a cellular state that responds accordingly to the extrinsic environment so that the tissue-resident cells and the tissue itself are capable of withstanding and adjusting to their environmental demands. However, the expression of *Mkx* and its downstream genes is load sensitive due to a complex and intricate network of mechanosensitive regulators which determine whether tenogenesis is maintained or whether chondrogenesis/osteogenesis is promoted. How *Gtf2ird1* and *Mkx* interact in this complex regulatory system remains to be elucidated.

### Future perspective.

The elucidation of the *Mkx*-regulating gene network may eventually lead to the direct reprogramming of a cell to a tenocyte. This has a significant therapeutic impact because tendon pathologies are long-standing, difficult to treat completely, and can be debilitating. Advances in tendon repair and development of bioartifical tissues have been slow due to the lack of molecular understanding. So far, the involvement of the TGF-β/Smad pathway has been implicated in tenogenesis (71, 72). *Scx*, which is heavily involved in tendon development, is also sensitive to mechanical cues, and although its role in tenogenesis seems to be independent of that of *Mkx*, further analysis is warranted (19, 20, 25, 26, 40). However, our findings indicate a significant role of *Mkx* in signal transduction that is independent of *Scx*, which may aid treatment of tendinopathy and identify optimal training conditions in athletes or rehabilitation programs postinjury to promote efficient tendon healing. Therefore, the *Mkx* gene is a potential therapeutic target for regenerative medicine and development of bioartificial tendons and ligaments.

In conclusion, Mkx, a tendon-specific transcription factor, is a mechanosensor that is transcriptionally induced by the binding of GTF2IRD1 to the promoter region through chromatin regulation. The nuclear translocation of Gtf2ird1 is provoked by cellular stretching, thereby linking mechanoforces to the Mkx-directed gene program that is essential for organized tendon development.

## Supplementary Material

Supplemental material
