# Supplementary material for: Gtf2ird1-Dependent Mohawk Expression Regulates Mechanosensing Properties of the Tendon
Source: Mol Cell Biol. 2016 Apr 1;36(8):1297–309. doi: 10.1128/MCB.00950-15 (PMC4836271; doi:10.1128/MCB.00950-15)
Supplement: Supplemental material [file supp_36_8_1297__index.html]

Supplemental material 

# *Gtf2ird1*-Dependent Mohawk Expression Regulates Mechanosensing Properties of the Tendon

## Supplemental material

- Supplemental file 1 -

  Fig. S1 (si*Gtf2ird1* efficacy), S2 (Exogenous GTF2IRD1 transfection distribution), and S3 (Cellular stretch-induced nuclear translocation in Mkx knockdown tenocytes)

  PDF, 4.4M
- Supplemental file 2 -

  Tables S1 (Primers for qRT-PCR), S2 and S3 (Deletion construct primers for luciferase assay [S2] and with TK promoter [S3]), S4 (Deletion of 68-bp sequence by inverse PCR), and S5 (ChIP primers)

  PDF, 128K
