# Supplementary material for: Gtf2ird1-Dependent Mohawk Expression Regulates Mechanosensing Properties of the Tendon
Source: Mol Cell Biol. 2016 Apr 1;36(8):1297–309. doi: 10.1128/MCB.00950-15 (PMC4836271; doi:10.1128/MCB.00950-15)
Supplement: Supplemental material [file MCB.00950-15_zmb999101200so1.pdf]

Supplementary Figure S1. si*Gtf2ird1* efficacy

A **qRT-PCR confirms si*Gtf2ird1* efficacy**

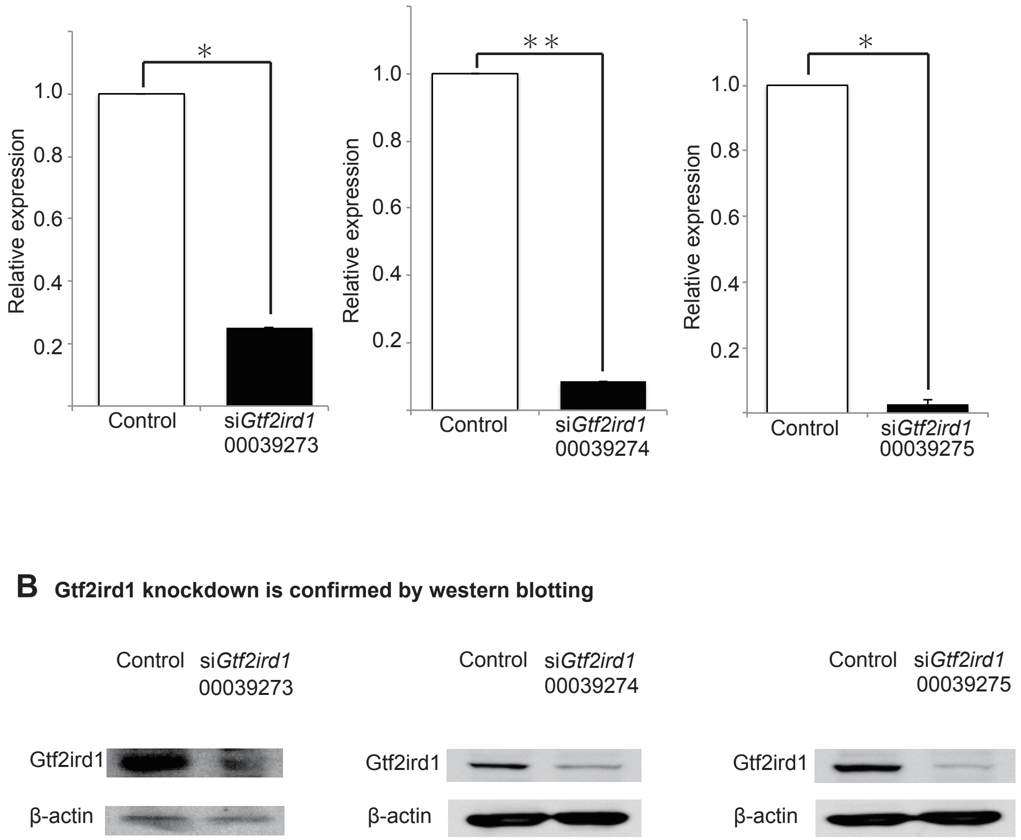

Supplementary Figure S2. Exogenous GTF2IRD1 is distributed in both cytoplasm and nucleus

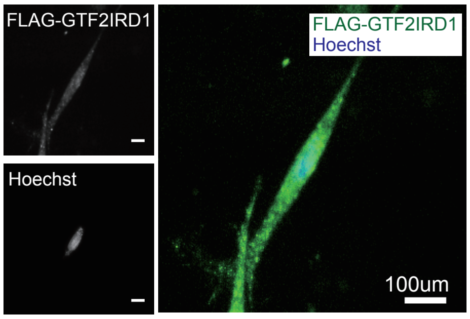

Supplementary Figure S3. Cellular stretch induces nuclear translocation in *Mkx* knockdown tenocytes

Subcellular localization of Gtf2ird1 with and without cellular stretch in *Mkx* knockdown cells.

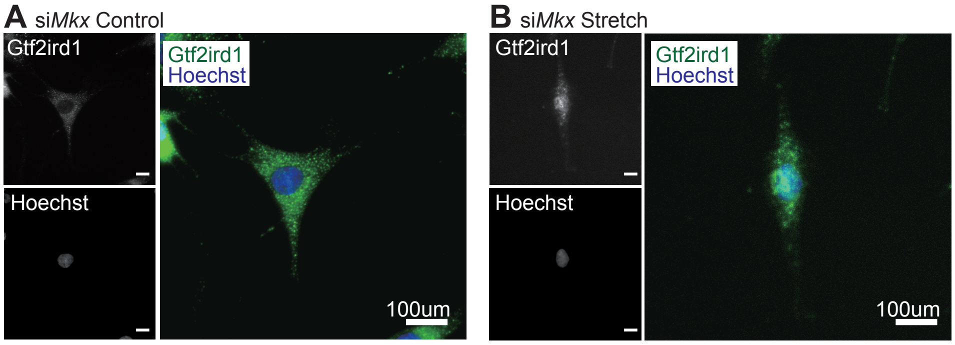

Supplementary Figure Legends.

Supplementary Figure S1. si*Gtf2ird1* efficacy.

(A) si*Gtf2ird1* efficacy was confirmed by qRT-PCR.

(B) si*Gtf2ird1* efficacy was also confirmed by western blotting. si*Gtf2ird1* (00039274) was selected for all si*Gtf2ird1* experiments in this article.

Supplementary Figure S2. Exogenous GTF2IRD1 transfection is distributed in both cytoplasm and nucleus.

Immunocytochemistry FLAG-GTF2IRD1 transfected tenocytes demonstrate a general induction of GTF2IRD1 both in nucleus and in cytoplasm. Anti-FLAG antibody was used as the primary antibody. (Green: FLAG-GTF2IRD1, Blue: Hoechst. Bars represent 100nm).

Supplementary Figure S3. Cellular stretch induces nuclear translocation in Mkx knockdown tenocytes.

(A) Subcellular localization of Mkx knockdown tenocytes demonstrate cytoplasmic localization of Gtf2ird1. (Green: Gtf2ird1, Blue: Hoechst. Bars represent 100nm)

(B) Subcellular localization of Mkx knockdown tenocytes demonstrate predominantly nuclear localization after stretch. (Green: Gtf2ird1, Blue: Hoechst. Bars represent 100nm)
