# Supplementary material for: Gtf2ird1-Dependent Mohawk Expression Regulates Mechanosensing Properties of the Tendon
Source: Mol Cell Biol. 2016 Apr 1;36(8):1297–309. doi: 10.1128/MCB.00950-15 (PMC4836271; doi:10.1128/MCB.00950-15)
Supplement: Supplemental material [file MCB.00950-15_zmb999101200so2.pdf]

Supplementary Tables

Supplementary Table 1. List of primers for qRT-PCR

Mouse

Gene name	Forward Primer	Reverse Primer
Gapdh	TTGTGGAAGGGCTCATGACC	TCTTCTGGGTGGCAGTGATG
Mkx	ACAATCCACACACAGGGCCG	GGTCTGCCGCCAGCTTTTATC
Gtf2ird1	TCGGATGTGTACCTGCTGCA	TCCCTGAGCAGCCTCTCATA
Scx	CCTTCTGCCTCAGCAACCAG	GGTCCAAAGTGGGGCTCTCCGTGACT
Tnmd	CTTTACCTCCACCAGGGCAACAGG	GCTTGATCTCGTTCCCATCCAG
Col1a1	GAGCGGAGAGTACTGGATCG	GCTTCTTTTCCTTGGGGTTC
Col1a2	CCAGCGAAGAACTCATACAGC	GGACACCCCTTCTACGTTGT
Fmod	CTTTACCTCCAGGGCAACAGG	GCTTGATCTCGTTCCCATCCAG
Lpl	AAGCTGGTGGGAAATGATGTGG	CCGTTCTGCATACTCAAAGTTAGG

Rat

Gene name	Forward Primer	Reverse Primer
Gapdh	AGACAGCCGCATCTTCTTGT	CCACAGTCTTCTGAGTGGCA
Mkx	GACGACGGCTGAAGAACATG	CCTCTTCGTTCATGTGAGTTCTTGG
Gtf2ird1	ATCTACCGAGTCCTGGCCAC	CCGTTGGTGGGGATGTCACA
Scx	CCCAAACAGATCTGCACCTT	CTTCAGTGGCTTCCACCTTC
Tnmd	CTACAGCAATGGCGAGAAGAAGAAG	GACCTACAAAGTAGATGCCAGTGTATC
Col1a1	GTCCGAGGTCCTAATGGAGATGC	GGTCCAGGGAATCCGATGT
Col1a2	CCTGGAGAACCTGGTCTCAT	GGCCAACATTTCCAGGAG
Fmod	CAAGGCAACAGGATCAATGAG	CTGCAGCTTGGAGAAGTTCA
Lpl	CTTCAACCACAGCAGCAAAA	TAGACTGGTTGTATCGGGCC

Supplementary Table 2. Deletion construct primers for luciferase Assay

luc 1 Fw Primer (+*Not*I)	AAAGCGGCCGCTGCATGTCGGCCATTTTAGC
luc 2 Fw Primer (+*Not*I)	AAAGCGGCCGCACCGAAGGCTTGTGAGATGC
luc 3 Fw Primer (+*Not*I)	AAAGCGGCCGCCTGTATCTCTTATGCGCGGC
luc 4 Fw Primer (+*Not*I)	AAAGCGGCCGCGGGGCACATGTAGATCCGAG
luc 5 Fw Primer (+*Not*I)	AAAGCGGCCGCCGTCTGAGATCTTGTGGGGG
luc 6 Fw Primer (+*Not*I)	AAAGCGGCCGGCCGGCTTGTTCCGATTCTCAC
Rv Primer	TTCGAGTGGGTAGAATGGCG

Supplementary Table 3. Deletion construct primers with TK promoter

del 1 Fw -663 (+*EcoR*V)	GATATCGGGGCACATGTAGATCCGAG
del 1 Rv -334 (+*Bgl*II)	AGATCTGCGGTGCAAATCCTGCGTTT
del 2 Fw -354 (+*EcoR*V)	GATATCAAACGCAGGATTTGCACCGC
del 2 Rv -25 (+*Bgl*II)	AGATCTCATGTTTCCCGACTGTCCGG
del 3 Fw -45 (+*EcoR*V)	GATATCCCGGACAGTCGGGAAACATG
del 3 Rv +282 (+*Bgl*II)	AGATCTCCCCACAAGATCTCAGACGG
del 4 Fw cons -519 (+*EcoR*V)	GATATCCTCCACAAGCCTGGACTTTG
del 4 Rv cons -373 (+*Bgl*II)	AGATCTAAATGTCACCAACCCAGGCC

Supplementary Table 4. Deletion of 68bp sequence by inverse PCR

68bp Deletion Fw	CCGACACTAGGAGCAGCTAA
68bp Deletion Rv	GCTGGAGGTTAGAGCTTCTT

Supplementary Table 5. ChIP primers

Primer site (kb)		Sequence
+2.0	Fw	TTATTCTGCTCCGCTGTCCT
	Rv	AATCAAGCTCCCACACCTCT
+0.9	Fw	AGGACTGGACTGGTGGAAAG
	Rv	AAAGCAAGTTTGGGAGCCTG
+0.2	Fw	AAATAAAGCCGAGACGACGC
	Rv	CTCAGGACTAGGCACACTCC
-0.3	Fw	AAGACACAGGATTTGCACCG
	Rv	TTCCTCTTGCGCTCAGAGAA
-0.6	Fw	CTGGGGTGCTTGTAGATCCA
	Rv	GGGAAAGGAGAGCTCAGGTG
-1.0	Fw	CCGGGAACAGCACAAAAGG
	Rv	GAACTCAGCAAGGCACGATT
-1.5	Fw	ACACAGCTCCGTAATCCAGT
	Rv	GTGCAACCTTCCCTTCTGAC
-1.8	Fw	TTAACACGGGCCCTCTACTG
	Rv	AGCACCTTAGTCACATGAGGT
-2.3	Fw	CCAAAAGCAACTCCCTCTACA
	Rv	AGCTAGGGACACATCAGAACA
-3.8	Fw	ATTGCACAAGGAAAGGAGGC
	Rv	TCTCCTTCCTCCCCTACCTC
-4.5	Fw	ACAGGGTAAGGATGGAGCAG
	Rv	AGAGAGCATATGTCGGTGGA
H1foo	Fw	TTGTTGTGGAAAGCGGAGTG
	Rv	AATGCAGATGGTTGGGCTTG
